# SPRAAKLAB - mobile laboratory for speech recorded acoustically and kinematically

**DOI:** 10.3758/s13428-025-02726-y

**Published:** 2025-06-20

**Authors:** Teja Rebernik, Jidde Jacobi, Raoul Buurke, Thomas B. Tienkamp, Defne Abur, Martijn Wieling

**Affiliations:** 1 Laboratoire de Phonétique et Phonologie, CNRS/Sorbonne Nouvelle, 4 Rue des Irlandais, Paris, 75005 France; 2 Brussels Centre for Language Studies, Vrije Universiteit Brussel, Pleinlaan 2, Brussels, 1050 Belgium; 3 Center for Language and Cognition Groningen, University of Groningen, Oude Kijk in ’t Jatstraat 26, Groningen, 9712 EK The Netherlands; 4 Department of Oral and Maxillofacial Surgery, University Medical Center Groningen, Hanzeplein 1, Groningen, 9700 RB The Netherlands; 5 Research School of Behavioural and Cognitive Neurosciences, University of Groningen, Antonius Deusinglaan 1, Groningen, 9713 AV The Netherlands

**Keywords:** Speech production, Speech perception, Mobile laboratory, Mobile data collection

## Abstract

Data collection in experimental linguistics is frequently conducted in laboratory rooms within a research institute, which can be difficult to reach for some participants, for example, those with mobility issues or living further away and in remote areas. This article presents SPRAAKLAB, a mobile laboratory that facilitates the collection of high-quality acoustic and articulatory data outside of university walls, thus bringing the laboratory environment closer to the participants. We present an acoustic analysis of recordings collected inside and outside of the SPRAAKLAB, including transmission loss, signal-to-noise ratio, and harmonics-to-noise ratio. All three measures reveal that the SPRAAKLAB is suitable for collecting consistent, high-quality speech data even in loud environments. Finally, we discuss how the SPRAAKLAB allows us to collect data more easily and facilitates public outreach activities.

## Introduction

To adequately capture linguistic diversity, experimental studies in linguistics should ideally include a large variety of speakers who differ in languages or dialects, ages, ethnicity, and socioeconomic and educational backgrounds. Despite this, participant samples of many studies are Western, Educated, Industrialized, Rich, and Democratic or WEIRD (Henrich, Heine, & Norenzayan, 2010). The tendency of linguistics studies to recruit “a homogenous population of compliant undergraduates” (Whalen & McDonough, 2015, p. 397) has been well-documented, with meta-analyses estimating that, for example, up to 88% of all adult samples in studies in applied linguistics consist of university students (Andringa & Godfroid, 2020).

At least some recruitment issues do not arise due to a lack of willingness to participate from the side of participants but rather due to more logistical issues. Many experimental studies are done within university walls (Kim, 2023). However, this is inconvenient when the goal is to recruit outside the university student pool and to include speakers who do not live close to the university or are less mobile, such as clinical populations or older dialect speakers. If only individuals who can and are willing to travel to a university can participate, the probability of introducing a selection bias increases.

Allowing people to participate from the comfort of their own homes can help make them more likely to participate. One way to achieve this is by collecting data online, which is possible for collecting acoustic data (Leemann, Jeszenszky, Steiner, Studerus, & Messerli 2020; Awan et al., 2023), although the quality of data collected online is highly dependent on the online recording software in use (Zhang, Jepson, Lohfink, & Arvaniti, 2021). Additionally, collecting data online is not always possible. For example, articulatory data must be collected in person (e.g., Lin, 2021). In addition, online data collection may present an additional barrier for older adults, hindering their participation.

Another solution is to use portable acoustic and articulatory recording equipment (Whalen & McDonough, 2015) and visit participants at their home or a nearby location (e.g., see Wieling et al., 2016 for an articulatory experiment, and see Strasser, Brand, & Rennies, 2022 for audio-visual experiments). In such cases, the participation threshold is lowered, but some disadvantages remain. First, each location will be different, and the location’s characteristics may affect the quality of the acoustic and articulatory recordings. The data that is collected using portable equipment may therefore not be entirely comparable across participants.^1^ Second, depending on the experimental paradigm, setting up the equipment can range from mildly complex (e.g., when collecting acoustic data requiring a microphone and a sound card) to very involved (e.g., when collecting articulatory data using electromagnetic articulography; EMA). Furthermore, setting up complex recording equipment can be very time-consuming. While setting up a portable electromagnetic articulograph is technically possible at each individual participant’s home, connecting and testing the many components may take up to an hour. This increases the probability of human error, even when following a checklist, and the possibility of damaging the expensive equipment.

A third solution is to bring a complete laboratory environment to the participant. While mobile laboratories are not common in linguistics, they have been used in audiology and to a lesser degree, psychology, for decades. Already in the 1960s, Chadwick (1963) described a mobile audiological unit. This truck consisted of an examination room, an audiometric booth, and a waiting room, and was intended for studying noise-induced hearing loss. Besides the mobile laboratory presented in this paper, only three mobile laboratories for collecting research data appear active in Europe. Two are built as a sound-isolated trailer, which can be towed by a car or van. One is the *Mobile Auditory Lab* of the Dutch Radboud University (Wassman, Janssen, & Agterberg, 2020), which was designed to study the perception of visual and auditory stimuli. The other is the *MobiLab* of the German RWTH Aachen University (Pausch & Fels, 2019), which was designed for on-site listening experiments. Finally, the *Manchester Voices Accent Van* (Drummond, Dann, Tasker, & Ryan, 2022) of the English Manchester Metropolitan University is a smaller van, intended for conducting sociolinguistic interviews on the go.

In this paper, we describe SPRAAKLAB, a mobile laboratory for speech recorded acoustically and kinematically. Our reasons behind building the SPRAAKLAB are twofold. First, we wished to be able to conduct high-quality data collection at speakers’ homes, as we frequently collect acoustic and kinematic data from older speakers, speakers with speech disorders and dialect speakers, for whom it is often more difficult to come to the university. Second, we are highly active in public outreach, which the SPRAAKLAB facilitates. The SPRAAKLAB thus needed to fulfil the relatively stringent criteria for our envisaged usage and specialized equipment (including e.g., electromagnetic articulography). It includes a custom-built cabin with an acoustically insulated room and is independent from a towing vehicle. This also makes it different from previous mobile laboratory implementations, which are either repurposed smaller vans without sound dampening (i.e., the Manchester Voices Accent Van) or towing trailers (i.e., the MobiLab and Mobile Auditory Lab).

The manuscript provides detailed information on the laboratory’s implementation, followed by an evaluation of the acoustic characteristics, including transmission loss, signal-to-noise ratio, and harmonics-to-noise ratio. We finally discuss some applications, including our practical experiences in running research projects using the laboratory. The appendices provide further information on the practical details of building the mobile laboratory (Appendix A) and our general setup during outreach events (Appendix B).

## Methods

### Implementation

The following sections describe the general setup of the mobile laboratory. More details on the procedure of constructing the mobile laboratory as well as its construction and maintenance costs can be found in Appendix A.

#### General

**Fig. 1:**
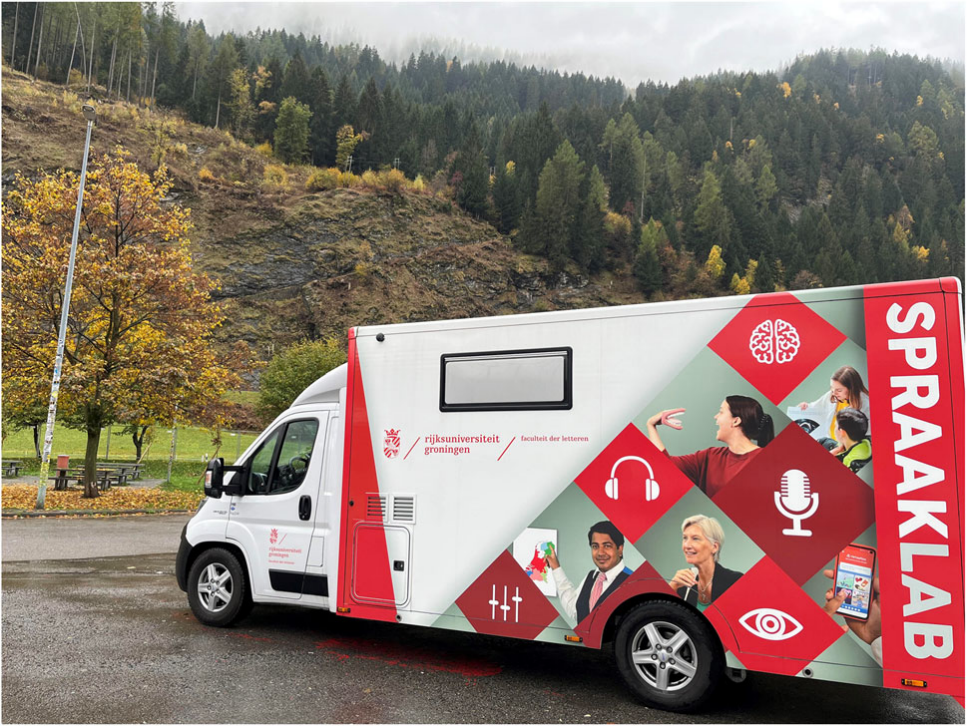
Outside design of the SPRAAKLAB on the driver’s side

The SPRAAKLAB consists of two parts: the front cabin (visible in Fig. 1) and the laboratory area at the back (see Section “Interior”, below). Its basis is a custom-made chassis with air suspension, attached to a Fiat Ducato 3.5T Heavy Chassis Cabin at the front. The laboratory area is a custom-built cabin. The total length of the mobile laboratory is 7 m, the width is 2.20 m (2.75 m with side-view mirrors), and the height is 2.95 m. The weight of the mobile laboratory is just below 3500 kg.

Due to the air suspension, the chassis can be electronically raised or lowered. By lowering the chassis, the laboratory is easier to step into, which is especially important when working with older adults or individuals with reduced mobility. This is additionally aided by an extendable step and a security handrail at the side of the door. Stabilizer legs are manually extendable and ensure stability while parked. While parked, the van needs to be attached to a power source. A power cable needs to be plugged into an available external power socket (rated 230V, 16A). More details on the outside of the SPRAAKLAB and its features ensuring traffic safety can be found in Appendix A.

**Fig. 2:**
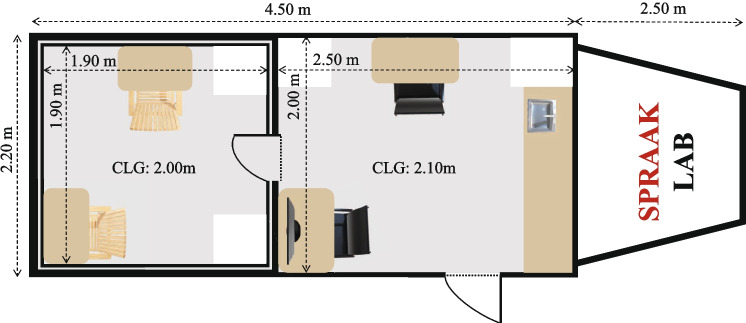
Floor plan of the SPRAAKLAB. CLG stands for ceiling height

#### Interior

The mobile laboratory space is separated into two rooms: the experimenter room and the acoustically insulated room, schematized in Fig. 2. The two rooms are separated by a sound-isolating door. The researcher(s) can observe the participant(s) through a one-way sound-isolated window positioned next to the door. An intercom system allows the researcher(s) in the experimenter room to communicate with the participant(s) seated in the acoustically insulated room. The mobile laboratory was designed to be flexible in terms of possible experimental setups. For example, dimmable lights, controlled separately for the two rooms, ensure optimal brightness levels for different types of experiments. Figure 2 shows the floor plan of the SPRAAKLAB.

**Fig. 3:**
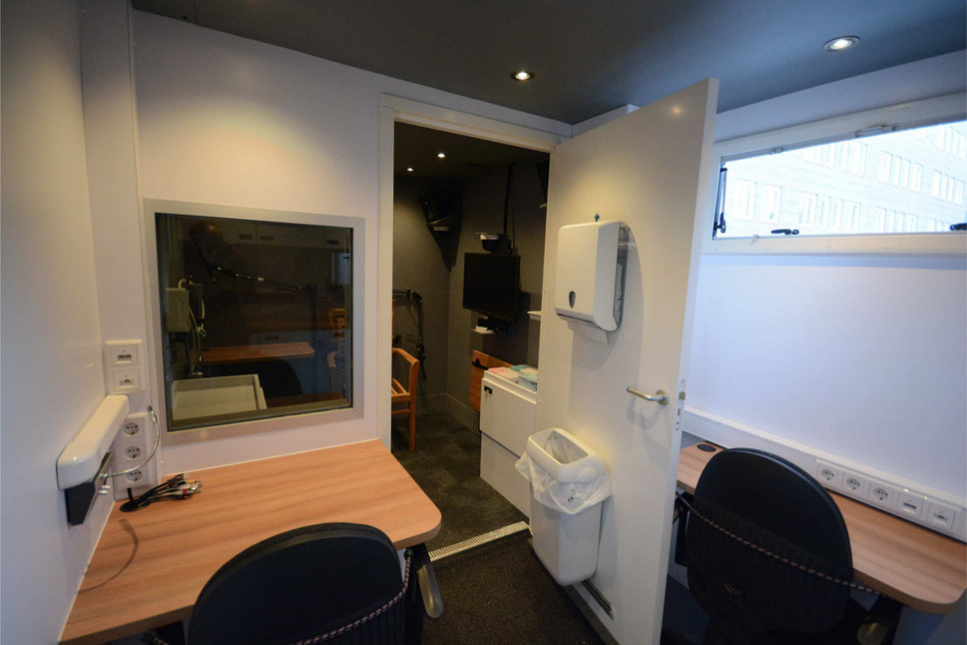
View from the experimenter room in the SPRAAKLAB. *From* *left to right*: intercom system, fixed desk with office chair, cables connected to PCs below the desk, wall with power plugs and Ethernet ports, one-way window providing a view of the participant, sound-dampened door with attached bin and paper towel dispenser, foldable desk with office chair, and wall with power plugs and Ethernet ports. See text and Appendix A for further details

##### Experimenter room

**Fig. 4:**
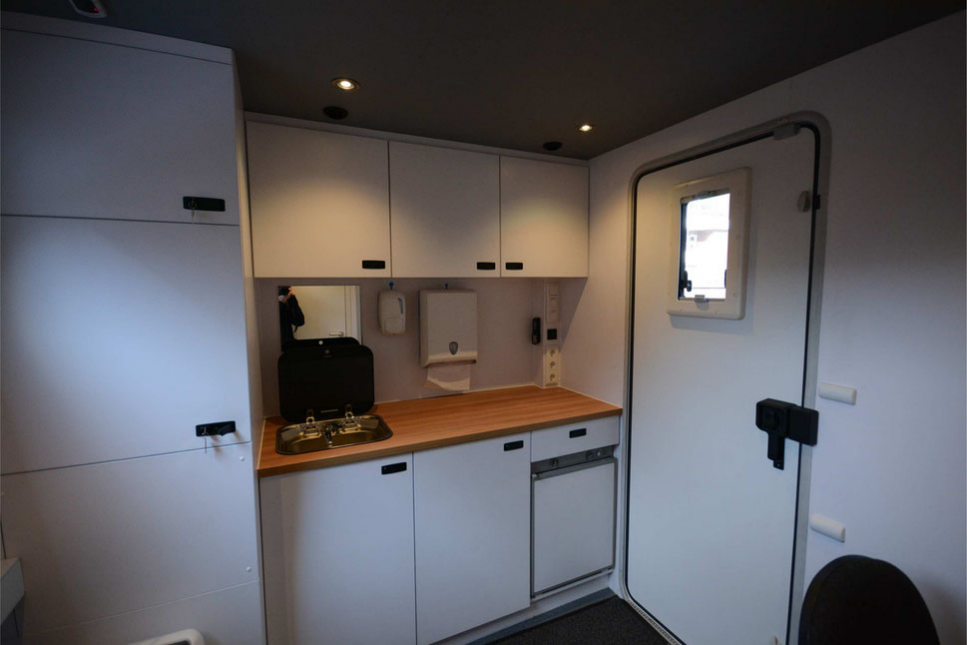
Kitchen in the SPRAAKLAB with a sink with mirror, soap, paper towel dispenser, and a mini fridge. See Appendix A for further details

The experimenter room, shown in Figs. 3 and 4, contains two desks (one fixed, one foldable), two office chairs, and a small kitchen. Beneath the fixed desk are two small high-performance PCs (HP Z2 mini G5), which control all audio, video, and articulatory recording equipment. Audio, video, and Ethernet cables are guided through sound-isolated ports in the walls to the acoustically insulated room. Portable monitors connected to the PCs can be placed on the fixed desk.

Several sound cards (including a TASCAM US-600 and Focusrite Scarlett Solo) and a sound-effects processor (Eventide Eclipse v4) are secured to the fixed desk to facilitate running multiple experiments with minimal cable rearrangement. The sound cards are connected to the microphones with eXternal Line Return (XLR) cables that run from the experimenter room to the acoustically insulated room. In addition to the experimental equipment, the experimenter room has a Network-Attached Storage (Synology DiskStation 1019+) with an uninterruptible power supply, connected to the PCs via Ethernet ports, ensuring a robust storage and backup system. For added comfort of the experimenters and the participants, the experimenter room also includes a heater and air conditioning (the intake and exhaust of the air conditioning unit are visible in Fig. 1). More details about the experimenter room can be found in Appendix A.

**Fig. 5:**
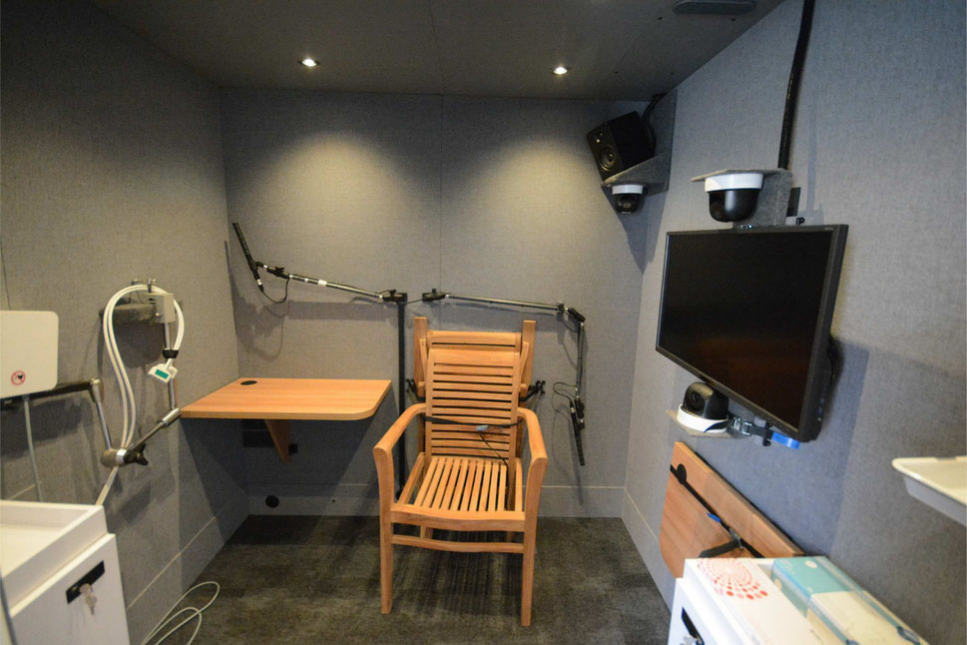
Acoustically insulated room in the SPRAAKLAB. *From left to right*: cupboard storing the NDI Vox-EMA and UTI systems, EMA field generator and sensor harnesses, first foldable desk (underneath which are the audio jack, slot with HDMI cable, power plugs, and Ethernet ports), two ME66 microphones on flexible arms, two wooden chairs (one sturdy, one foldable behind), left-most audio speaker, monitor on flexible arm, three pan-tilt-zoom cameras (upper left corner of the room, below and above the monitor), second foldable desk, and cupboard (above which is a disinfection dispenser, partly visible)

**Fig. 6:**
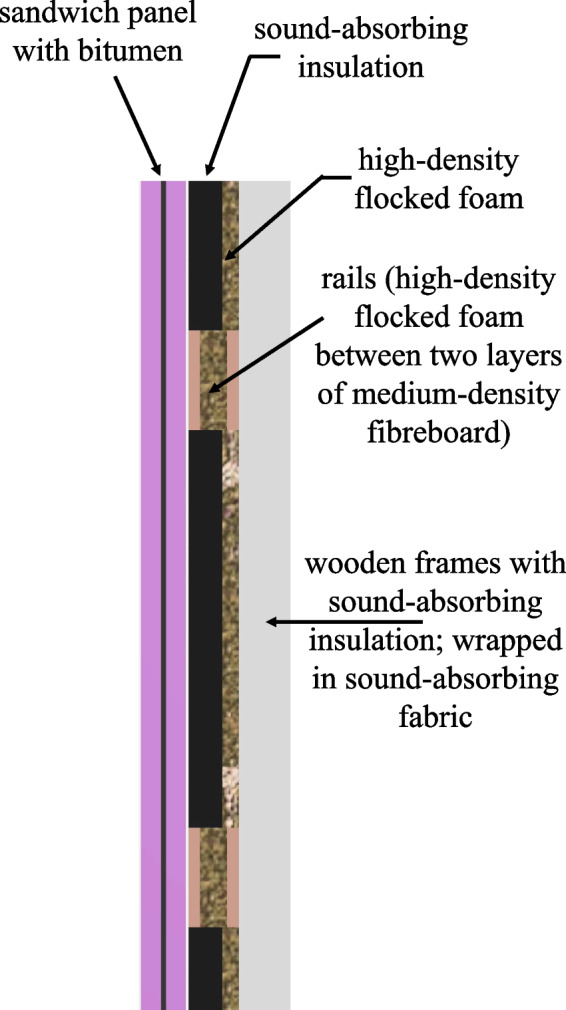
Cross section of the sound-dampening wall

##### Acoustically insulated room

The acoustically insulated room, depicted in Fig. 5, reduces outside sound by 30 dB SPL (see also Subsection “Results”). The room has been sound-dampened using four layers combining bitumen, wood, and dampening foam. A cross section of the wall is shown in Fig. 6.

The acoustically insulated room has two foldable desks and two sturdy wooden chairs (one foldable), which enables us to run experiments with either a single participant or a participant pair. Visual stimuli can be presented on a 32” computer monitor, which is attached to an extendable flexible VESA arm that can also be adjusted vertically and horizontally. Portable monitors can additionally be connected. Two directional microphones (Sennheiser ME66) are attached to flexible arms, which can reach all places where participants can be positioned. If needed, the XLR cable can be connected to a headset microphone. Both desks have auxiliary (AUX) ear/headphone slots underneath them, and there is an additional stereo speaker system, which can be connected via Bluetooth or an AUX cable and controlled from the experimenter room. As we carry out many articulatory kinematic experiments, a shelf for the arm of our NDI-VOX EMA system (see Rebernik, Jacobi, Tiede, & Wieling, 2021) was added, as well as a designated cupboard for the EMA and ultrasound tongue imaging (UTI) equipment.

Finally, the acoustically insulated room contains three pan-tilt-zoom IP cameras, which are attached above and below the 32” monitor and in the outer top-left corner of the room. The view of the cameras can be monitored from the computers in the experimenter room. The camera positioned in the top-left corner provides a clear overview of the acoustically insulated room. The cameras above and below the monitor are useful for kinematic speech production experiments. Specifically, the camera above the monitor can be used to observe the participant’s mouth, which is especially useful during experiments with EMA, when sensors are attached to the tongue, the lips, and the jaw. The camera below the monitor can be used to observe whether the UTI probe remains in a stable position below the chin of the participant. During some kinematic experiments, recordings are made with the cameras to verify data quality after data collection has ended.

### Evaluation

#### Objectives

The aim of the evaluation was to determine the suitability of the SPRAAKLAB for acoustic data collection and analysis. In order to do so, we conducted two sets of measurements. First, we measured sound pressure levels (SPL) at a festival location to determine the effective amount of sound-dampening provided by the acoustically insulated room. Second, we compared the signal-to-noise ratios (SNR) and harmonics-to-noise ratios (HNR) of audio recordings made in typical and adverse recording environments to assess recording quality.

#### Equipment and procedure

Using a shotgun microphone (Audio Technica AT875R), digitized via a soundcard (Focusrite Scarlett Solo) at a 44.1-kHz sampling rate, we recorded a 3-s sample of sustained phonation and a 20-s speech sample, preceded by 10 s of silence.

For the speech samples, a 26-year-old male speaker (paper co-author T.B.T) was instructed to either speak at his habitual volume or a loud volume: this accounted for the fact that in loud environments, speakers will unintentionally raise their voice to remain understood (i.e., the so-called Lombard effect; Zollinger & Brumm, 2011). We recorded both loud and habitual conditions at multiple locations (see below), using the same gain, and equal microphone-to-mouth distance, which was verified with a ruler (about 10 cm).

Due to speaker variability, we additionally recorded a pre-recorded audio sample of the same speaker, played via in-built smartphone speakers (Samsung S22) at a set maximum loudness level and a distance of 10 cm from the microphone. This ensured that we could reliably compare signal-to-noise and harmonics-to-noise ratios (see Section “Results”) captured inside and outside of the SPRAAKLAB.

#### Recording locations

There are two main sites where we use the mobile laboratory: at people’s homes and festivals. To test the sound-dampening properties of the SPRAAKLAB, we therefore conducted the aforementioned sets of measurements across multiple conditions across different environments. Specifically, we made 12 recordings in the acoustically insulated room of the SPRAAKLAB. Four of these were recorded in a normal non-noisy environment (i.e., university parking lot) and eight in a noisier festival environment. For comparison purposes, we also made 12 recordings outside of the SPRAAKLAB. Four of these were made in an acoustic booth (Studiobricks Double Wall) at the university, while eight were made at the same festival directly outside of the SPRAAKLAB.

For the recording of pre-recorded audio samples, we collected five recordings at a festival inside the SPRAAKLAB, five outside of the SPRAAKLAB, two inside the SPRAAKLAB in a non-noisy environment, and two inside the university acoustic booth.

#### Metrics

To determine the amount of sound dampening (hereinafter: “transmission loss”), we measured the SPL using a handheld SPL meter (Digital Sound 8922, Az Instrument Inc.) with A-weighting to mimic the response of the human ear and the default “fast” time weighting. At festival grounds, a researcher took 10-s measurements in the acoustically insulated room inside the SPRAAKLAB, and directly outside of the SPRAAKLAB, on three separate days, with the SPL meter set to “max hold” (to measure the highest detected SPL).

To determine the quality of acoustic recordings made in the SPRAAKLAB, we calculated the signal-to-noise (SNR) and harmonics-to-noise (HNR) ratios of recordings made inside the acoustically insulated room of the SPRAAKLAB, and compared them to recordings made outside of the SPRAAKLAB (in an acoustic booth and at the festival). SNR is a measure comparing the power of the desired signal (in our case: speech) to the power of the noise. The louder the noise with respect to the signal, the lower the ratio. It is generally considered that an SNR ratio above 30 dB is acceptable for acoustic analysis, but an SNR ratio above 42 dB is recommended (Deliyski, Shaw, & Evans, 2005).

Using PRAAT (Boersma & Weenink, 2022), we extracted the maximum absolute pressure of the entire signal ($p_{signal}$) and the maximum absolute pressure of a representative portion of silence ($p_{noise}$). We then calculated the SNR using the following formula (see also Svec & Granqvist, 2018):

1 $$\begin{aligned} SNR = 20 * log(p_{signal} / p_{noise}) \end{aligned}$$

HNR is a measure that compares period and non-periodic (i.e., harmonic and noisy) components of a speech signal. In Praat, we extracted the sustained phonation part of the recording by skipping the first 500 ms and last 500 ms of the vowel. Using the “To Harmonicity (cc)” function and default settings, we extracted the harmonicity object and then used “Query - Get mean” to obtain the mean HNR for the sustained vowel of each recording.

## Results

Table 1 shows the transmission loss, mean SNR (SD in parentheses), and mean HNR (SD in parentheses) for each location, including recordings collected with a speaker (habitual versus loud speech) and recordings collected with a pre-recorded speech sample.

**Table 1:** Sound pressure level (SPL in dB(A)), signal-to-noise ratio (SNR) and harmonics-to-noise ratio (HNR) for all conditions and locations

Location	SPL	SNR	HNR
**Speaker: habitual**			
SPRAAKLAB (normal)	34.5	49.5	18.8
Acoustic booth (normal)	33.7	50.2	18.4
SPRAAKLAB (noisy)	38.9 (2.4)	42.7 (3.7)	19.0 (0.6)
Festival (noisy)	72.2 (4.3)	34.5 (2.7)	17.2 (1.2)
**Speaker: loud**			
SPRAAKLAB (normal)	-	52.8	20.1
Acoustic booth (normal)	-	49.3	19.7
SPRAAKLAB (noisy)	-	46.8 (3.4)	20.5 (0.9)
Festival (noisy)	-	35.8 (2.1)	21.7 (1.3)
**Pre-recorded sample**			
SPRAAKLAB (normal)	-	44.7	6.8
Acoustic booth (normal)	-	44.1	7.5
SPRAAKLAB (noisy)	37.0 (1.7)	40.4 (2.3)	7.0 (0.4)
Festival (noisy)	71.0 (4.1)	20.3 (3.7)	3.8 (1.6)

Where only two recordings are available, there is no standard deviation (SD) provided, only the average of the two values. SPL for speaker recordings is the same for habitual and loud speech

### Transmission loss

Transmission loss assessment (column “SPL” in Table 1 revealed that the SPRAAKLAB’s acoustically insulated room dampens outside sound by 33.4 dB SPL on average (standard deviation [SD]: 1.9 dB). As we frequently use the air conditioning unit during the summer and it was on during the recordings reported in Table 1, we took additional recordings of ambient noise in the acoustically insulated room with the air conditioning unit turned off and on. These recordings were done in a quiet environment, at the parking lot of our university building. The average ambient noise was 39.6 dB SPL (SD: 0.1 dB SPL) with the air conditioning turned off, and 41.8 dB SPL (SD: 0.5 dB SPL) with the air conditioning unit turned on.

### Harmonic-to-noise ratio

**Fig. 7:**
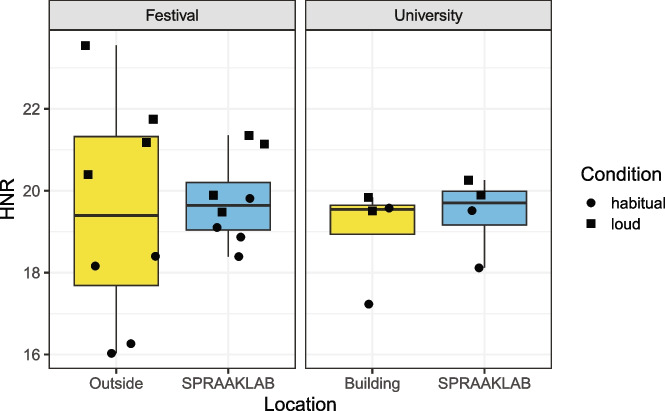
Harmonics-to-noise ratio per location and condition

Figure 7 visualizes the HNR values for each location using box plots, separated per location (festival vs. university) and condition (habitual vs. loud speech).

### Signal-to-noise ratio

**Fig. 8:**
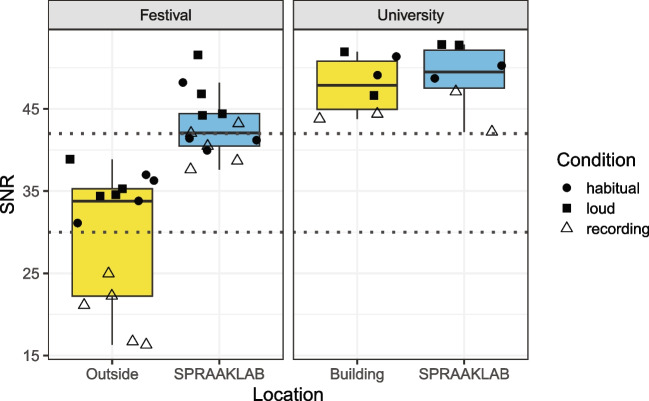
Signal-to-noise ratio per location and condition. The *dashed lines* represent the recommended SNR of 42 dB and the acceptable level of 30 dB

Figure 8 visualizes the SNR values for each location using box plots, separated per location (festival vs. university) and condition (habitual vs. loud speech).

## Discussion

### Metrics

We used three metrics to evaluate the SPRAAKLAB’s performance in two types of environments (normal and noisy) and in multiple conditions (habitual speech, loud speech, and pre-recorded speech sample). The first metric, transmission loss (measured as SPL) with a handheld SPL meter, revealed that the SPRAAKLAB has an effective sound-dampening of around 30 dB in loud environments. The ambient noise levels of the SPRAAKLAB (around 35–40 dB) are comparable to those of another mobile laboratory, *MobiLab*, which were reported to be between 20-40 dB at active school yards (Pausch & Fels, 2019). Another mobile laboratory used specifically for sociophonetics research, *Manchester Voices Accent Van* (Drummond et al., 2022), is a smaller van without an acoustically insulated room. While the authors do not report ambient noise levels, they have noted that the recordings created at louder public events included relatively high degrees of background noise (Drummond et al., 2022, pp. 274).

The second metric we included, HNR, measured on the basis of a sustained vowel, was shown to be more variable in a noisy outside environment, and comparable between the SPRAAKLAB and an acoustic booth. Overall, the HNR values recorded for the speaker fell within normative range for young male speakers for recordings made in the SPRAAKLAB and the acoustic booth, but not all recordings made outside (Goy, Fernandes, Pichora-Fuller, & van Lieshout, 2013).

The SNR, measured on the basis of the entire recording, showed that the recording environment inside the SPRAAKLAB is comparable to that of an acoustic booth inside a university building. In a noisy environment, the SNR deteriorates slightly but overall remains at the recommended SNR level above 42 dB and well above the acceptable 30 dB threshold.

The three metrics we included provide evidence that acoustic recordings inside the SPRAAKLAB are of comparable quality to acoustic recordings in a sound booth.

### SPRAAKLAB application examples

There are multiple ways in which SPRAAKLAB is useful at our work. One advantage is that it allows us to collect data close to participants’ homes. This lowers the participants’ time investment, thus making them more likely to participate in our studies.

For example, one project in our lab used the mobile laboratory for a large-scale dialect data collection, conducted in 2022 and 2023 (Buurke, Tienkamp, Heeringa, Knooihuizen, & Wieling, submitted). This study investigated regional language change in the northern and eastern Netherlands. Using the mobile laboratory, the researchers could include more than 100 dialect speakers across 32 sampling locations in five Dutch provinces. For that study, a second advantage of the mobile laboratory was that the separate experimenter and acoustically insulated room mitigated the so-called “observer’s paradox”. This paradox remains a notoriously difficult problem to solve (Cukor-Avila, 2000; Feagin, 2013), as dialect speakers are more prone to use formal speech norms (e.g., standard language instead of dialectal speech) when a researcher is present. Therefore, a physical separation between the two rooms helps to collect more authentic dialect data.

Another project that used the SPRAAKLAB investigated the speech of individuals with lateral tongue carcinomas before and after surgery using EMA (Tienkamp et al., 2024). After the diagnosis and determining the best treatment modality, the patient’s surgery was scheduled as quickly as possible. This typically resulted in a period of only seven to 10 days to schedule an appointment with a patient, during which they also had many other clinical appointments. Without the mobile laboratory which allowed for testing the participants at their homes, recruitment would have been very challenging. Additionally, the mobile laboratory also benefited from a project on speech motor control in Parkinson’s disease. In that project (see e.g., Hoekzema et al., 2024), the SPRAAKLAB enabled us to reach older individuals and individuals with advanced symptoms of Parkinson’s disease. As many individuals with Parkinson’s disease suffer from fatigue, the mobile laboratory ensured that they were able to complete the experimental sessions without unnecessary travel time and that they were willing to participate in a study consisting of multiple sessions (i.e., none of the 64 individuals dropped out during the study). We also completed the initial paperwork with the participants in the comfort of their own homes.

Another advantage of the mobile laboratory is that it can be employed at public engagement (i.e., outreach) events. These can either be fully focused on science outreach (in a broad sense), or they are more general events with a minor focus on science outreach. In the former category, we have attended local events in the Netherlands (e.g., *Expeditie NEXT*), but also abroad (e.g., *Festival della Scienza* in Genoa, Italy). In the latter category, we have attended some of the largest music festivals in the Netherlands, including *Lowlands* and *Zwarte Cross*. At all events, we provide multiple activities aimed at teaching the general public how speech works and promoting regional language appreciation and awareness. In our activities, we try to follow best practices in scientific communication and outreach. Specifically, we abide by the AEIOU definition of science communication (Burns, O’Connor, & Stocklmayer, 2003) in raising Awareness, Enjoyment, Interest, helping people form Opinions, and encouraging Understanding.

In addition to demonstrating research techniques (see Appendix A for details) and increasing the visibility of the university, we also use the SPRAAKLAB to obtain research data. Specifically, we generally try to collect speech data in short experiments^2^ ranging from five to 20 min. For example, in only three consecutive days in the summer of 2022, we collected formant perturbation data from 30 Dutch children in a very short 5-min experiment conducted at the Dutch festival *Zwarte Cross* (Rebernik et al., 2023). At the subsequent edition of *Zwarte Cross*, we collected acoustic dialect data from over 100 participants living all across the Netherlands. At the cultural festival *Noorderzon* in Groningen, the Netherlands, in 2022, we collected UTI data (likewise in a 5-min experiment) on the production of liquids in a sample of 70 adult participants during six consecutive days (Tiede, Boyce, Stern, Rebernik, & Wieling, 2024). In the 2024 edition of *Noorderzon*, we collected speech recordings and perceptual judgments of Dutch regional accents from over 300 participants in less than two weeks. Finally, at the *Festival della Scienza* in Genoa, Italy, in October 2022, we collected formant perturbation data in a 5-min experiment from over 90 Italian native speakers during a single week (Polsterer et al., 2023).

### SPRAAKLAB disadvantages

There are also some disadvantages associated with the mobile laboratory. While the participants do not have to host us inside of their house, they do in most cases need to provide us with a parking spot and an electrical outlet. This is easier for people who live in a house than those who live in an apartment. Additionally, due to the size of the mobile lab, some locations are still more easily reachable than others. It is, for example, not easy to set up in front of apartment buildings or in a city center. In those cases, nearby public locations (e.g., large parking lots of shopping centers, schools, or hospitals) are generally used instead. Accessibility to the mobile laboratory is furthermore limited. While there is an extendable step and handrail at the entrance, the mobile laboratory is not accessible for people with severely reduced mobility, such as wheelchair users, due to a relatively narrow entrance door of $\sim$70cm and the absence of a ramp.

Another potential disadvantage is the mobile laboratory’s visibility. While this is an asset when conducting public engagement activities, it could be considered a drawback when visiting participants. Given the outside design of the van, it is immediately clear that researchers are visiting to conduct speech research. Depending on the population in question, this could be considered too revealing. For example, visiting an individual with Parkinson’s disease might prompt the neighbors to become aware of an individual’s speech problems. So far, participants have not voiced this concern. Should a participant worry about the visibility of the mobile laboratory, this can also easily be solved by parking it at a nearby public location.

Finally, a substantial disadvantage to the researchers using it is the size and complexity of the mobile laboratory. Before using and driving the mobile laboratory, the researchers first need to become acquainted with driving a vehicle of this size and weight to limit the risk of damage due to an accident. They also need ample training to be able to use and set up all equipment from scratch before data collection can start. From the researchers’ perspective, therefore, there is a large initial time investment necessary before one can start using the mobile laboratory effectively.

## Conclusion

The goal of this paper was to introduce the SPRAAKLAB, the mobile laboratory of the Faculty of Arts of the University of Groningen. We provided the specifications of the mobile laboratory and evaluated its suitability for collecting acoustic recordings. Our evaluation revealed that the SPRAAKLAB performs comparably to an acoustic booth when located in a quiet environment, and can still be used to collect acceptable acoustic data when located in a loud environment. Finally we discussed some application examples for the laboratory, which we hope demonstrate its broad usefulness.

## Figures and Tables

**Fig. 9:**
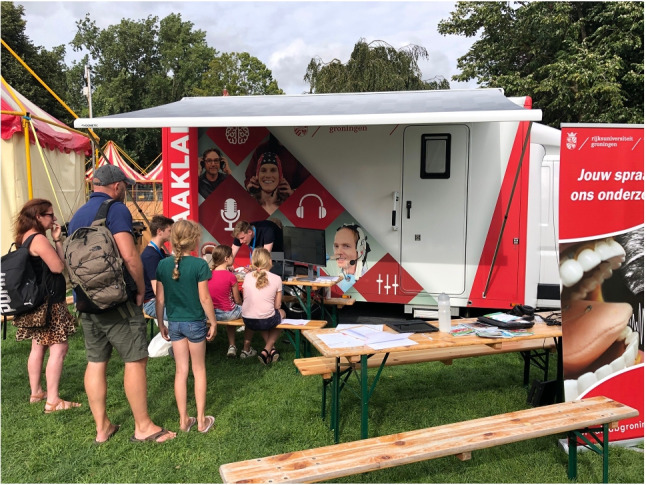
A common setup at events we visit. This side of the mobile laboratory also shows the awning and access door. See Fig. 1 for a view of the other side

## Data Availability

The data that has been used to report the SNR and HNR of the SPRAAKLAB can be found at https://github.com/tejarebernik/spraaklab.

## Appendix A: Practical details

The following sections provide more practical details about the SPRAAKLAB, including a detailed list of costs that were involved in creating the mobile laboratory (Subsection “Costs of building and maintaining a mobile laboratory”) and information on the furnishing and equipment (Subsection “Equipment”).

### A.1: Costs of building and maintaining a mobile laboratory

At the beginning of 2020, the board of the University of Groningen offered a grant of €150,000 to the Faculty of Arts to purchase a custom-made mobile laboratory. The chassis of the mobile laboratory was built by Coxx Mobile System B.V. (Rijen, The Netherlands), while the construction of the laboratory space itself was designed and created by Carrosserie Akkermans B.V. (Oud Gastel, The Netherlands). Construction began in April 2020 and was completed in January 2021.

The mobile laboratory cost around €150,000 to build, including the following costs rounded to the nearest €100: chassis (€14,100), Fiat Ducato Maxi (€27,000), construction of the mobile laboratory (€107,400), and road safety approval costs (€1,300). In addition, €35,000 was spent on additional hardware and equipment.

The annual costs of maintaining a mobile laboratory amount to around €15,000 and include the costs of diesel fuel, insurance, roadside assistance, road tax, an alarm system subscription, annual maintenance costs, and periodic technical inspections and repairs.

### A.2: Equipment

#### A.2.1: General equipment and design

A locked storage area, visible in Fig. 1, can be accessed from the outside. It is used to store a generator (Honda EU 22i), which serves as backup power, as well as two extension power strips with multiple sockets and a 50-m extension cable (the latter lending further flexibility to experimental setups). The locked storage area also contains two power sockets. These can be used to plug in two power strips which can be guided through a closable hatch in the floor of the storage area to ensure power can be made available outside of the van.

Due to our frequent outreach activities (see Appendix B on ‘Public engagement’), some features have been added to protect equipment we regularly use outside in various weather conditions. Specifically, a large awning above the door provides shade (see Fig. 9 in the main text), and side walls and a front panel can be attached to the awning to protect against wind, sun, and rain.

The mobile laboratory is equipped with multiple safety features. To ensure traffic safety, there is a 360-degree bird-eye camera system, with the view displayed on a small monitor inside the driver’s cabin. Inside the cabin is also a digital rear-view mirror and a rear cross-traffic collision warning system, which detects objects when driving in reverse. In addition, when driving in reverse, the mobile laboratory emits a loud beeping sound, warning pedestrians and cyclists. The available safety features also include elements that protect the mobile laboratory against theft and vandalism.

The outside design of the van displays various techniques used in linguistics research at our faculty, amongst others including ultrasound tongue imaging, electromagnetic articulography, acoustic data collection and electroencephalography.

#### A.2.2: Specific equipment

The equipment and furnishing in the experimenter room and acoustically insulated room are listed in Table 2. Everything listed always needs to be safely secured for driving.

**Table 2:** List of furniture, equipment and other amenities in the experimenter room (*left column*) and acoustically insulated room (*right column*)

Experimenter room	Acoustically insulated room
**Equipment**	
Two PC’s (HP Z2 mini G5)	NDI-Vox EMA
Intercom system	NDI-Vox EMA attachment arm
Sound cards (TASCAM US-600,	UTI (Telemed probe)
Focusrite Scarett Solo)	Two directional microphones
Audio splitter	Stereo speaker system
Sound-effects processor	32” computer monitor
(Eventide Eclipse v4)	
Portable monitors	
Synology DS1019+ NAS	
NAS power supply	
**Furniture and amenities**	
Two desks (one fixed, one foldable)	Two foldable desks
Two office chairs	Two sturdy chairs (one foldable)
Multiple cupboards	Multiple cupboards
Power sockets	Power sockets
Ethernet ports	Ethernet ports
Small portable toilet	Three pan-tilt-zoom IP cameras
Air conditioning	Two headphone sockets
Heater	Two microphone sockets
Fire extinguisher	
**Kitchen**	
Sink and cold water tap	
Mirror	
Small refrigerator	
Coffee machine	
Kettle	

Abbreviations: EMA (Electromagnetic Articulography), UTI (Ultrasound Tongue Imaging), NAS (Network Attached Storage)

## Appendix B: Setup during outreach events

At events, we set up tables and chairs in front of the mobile laboratory, underneath the awning (see Fig. 9 for a common setup). We organize various activities for children and adults, aimed at teaching them about how speech works and explaining what kind of research we do. For example, we explain the basics of speech motor control by showing people the movement of their tongue using UTI (after which they receive a printout of the ultrasound image as a keepsake) and teach them about speech anatomy using a physical model of the vocal tract. The visitors can generate vowel sounds by using an electrolarynx in combination with a sliding vocal tract model (Arai, 2016). Finally, we also provide a short speech quiz on a tablet and some word search puzzles.

We also promote regional language appreciation and use in various ways. For example, we demonstrate an app aimed at teaching primary school children the regional language (Low Saxon varieties) and an app that predicts where someone originates from on the basis of the dialectal variants they indicate to use (similar to Hilton, 2021). We have also developed a popular experiment where an AI system tries to predict where someone originates from on the basis of their (Dutch) accent.^3^ Furthermore, we showcase an educational dialect board game, in which trivia questions about linguistics and regional languages in the Netherlands and Flanders need to be answered in order to progress in the game.^4^

## Footnotes

1 Note that we refer specifically to issues due to variability that arises because of differences across locations. Other sources of variability can stem from participant performance, for example participants failing to perform the experiments according to instructions or making general mistakes.

2 For all experiments, approval from our faculty’s Research Ethics Review Committee is always obtained beforehand, and adults (in the case of children: their caretakers) give written informed consent before participating.

## Code Availability

The code used to generate Figs. 7 and 8 can be found at https://github.com/tejarebernik/spraaklab.
